# Sex steroid hormones and allergic diseases in children: a pilot birth cohort study in the Japan Environment and Children’s Study cohort

**DOI:** 10.1186/s12887-023-04302-9

**Published:** 2023-09-21

**Authors:** Yumiko Miyaji, Kiwako Yamamoto-Hanada, Limin Yang, Mayako Saito-Abe, Miori Sato, Hidetoshi Mezawa, Minaho Nishizato, Masayuki Ochiai, Shouichi Ohga, Masako Oda, Hiroshi Mitsubuchi, Masayuki Shimono, Reiko Suga, Nathan Mise, Makiko Sekiyama, Shoji F. Nakayama, Yukihiro Ohya, the Japan Environment and Children’s Study (JECS)

**Affiliations:** 1 Allergy Center, National Center for Child Health and Development, 2-10-1 Okura, Setagaya-Ku, Tokyo, 157-8535 Japan; 2 Medical Support Center for the Japan Environment and Children’s Study, National Research Institute for Child Health and Development, 2-10-1 Okura, Setagaya-Ku, Tokyo, 157-8535 Japan; 3 Department of Pediatrics, Graduate School of Medical Sciences, Kyushu University, Fukuoka, Japan; 4 Research Center for Environment and Developmental Medical Sciences, Kyushu University, Fukuoka, Japan; 5 The South Kyushu Okinawa Unit Center, Faculty of Life Sciences, Kumamoto University, Kumamoto, Japan; 6 Department of Neonatology, Kumamoto University Hospital, Kumamoto, Japan; 7 Regional Center for Pilot Study of Japan Environment and Children’s Study, University of Occupational and Environmental Health, Fukuoka, Japan; 8 Department of Environmental and Preventive Medicine, Jichi Medical University, Tochigi, Japan; 9 Japan Environment and Children’s Study Programme Office, National Institute for Environmental Studies, Ibaraki, Japan

**Keywords:** Allergic disease, Asthma, Atopic dermatitis, Dehydroepiandrosterone sulfate, Follicle-stimulating hormone, Sex steroid hormone

## Abstract

**Background:**

Numerous studies suggest that sex steroids might play a role in sex disparity observed in allergic diseases in adults. However, whether sex hormones influence allergic diseases in children remains unclear. The aim of the present study was to examine the association of sex steroid hormones with allergic disease in Japanese children.

**Methods:**

The present cross-sectional study included 145 6-year-old children participating in a pilot birth cohort study in the Japan Environment and Children’s Study. Data on allergic diseases were obtained from questionnaires, and serum levels of sex steroid hormones and allergen-specific IgE were measured. Logistic regression was performed to evaluate the association of sex hormones with allergic diseases.

**Results:**

After adjusted sex, amount of body fat at 6 years, parental history of allergic disease, and exposure to tobacco smoke, serum dehydroepiandrosterone sulfate level was significantly associated with reduced odds of any allergic disease (adjusted odds ratio, 0.58; 95% confidence interval, 0.36–0.93; *P* = 0.024) and serum follicle-stimulating hormone level was significantly associated with increased odds of any allergic disease (adjusted odds ratio, 2.04; 95% confidence interval, 1.01–4.11, *P* = 0.046). Dehydroepiandrosterone sulfate level showed a significant association with number of allergic diseases.

**Conclusions:**

The current study findings suggest that sex hormones may play an important role in the development of allergic diseases in prepubertal children.

**Supplementary Information:**

The online version contains supplementary material available at 10.1186/s12887-023-04302-9.

## Background

Several longitudinal studies have reported sex differences in allergic diseases [1–5]. Before puberty, boys are more likely to be diagnosed with allergic disease and sensitization and are more likely to use medications for allergic diseases compared to girls [1, 2]. A shift in sex distribution of allergic disease prevalence is observed after puberty, and allergic diseases become more common and severe or more treatment-resistant in females than in males [3]. Specifically, numerous epidemiologic studies have reported sex-specific differences in asthma [1, 2]. The Prevention and Incidence of Asthma and Mite Allergy study reported that the cumulative incidence of parent-reported asthma was higher in boys than in girls (15.1% vs. 10.8%) at 8 years of age [3]. The TRacking Adolescents’ Individual Lives Survey study reported that the prevalence of asthma was similar between boys and girls (7.7% and 7.4%, respectively) at a mean age of 11.1 years and that the prevalence of asthma was significantly higher in females than in males (6.2% vs. 4.3%) at 16.3 years of age [4]. These studies indicate that sex differences in allergic diseases begin early in childhood, with a shift in prevalence occurring in puberty.

These sex disparities suggest that sex steroids might play a role in allergic diseases. A recent systematic review and meta-analysis of 57 studies on asthma, including 51 observational and 6 experimental studies, reported that more estrogenic state such as, menstruation, menopause onset, and hormonal replacement therapy, in females were associated with the increased risk of current and new-onset asthma [5]. Specific mechanism underlying the impact of sex hormones on allergic diseases in children is unclear.

The aim of the present study was to examine the association of sex steroid hormones with allergic diseases in Japanese children.

## Methods

### Study design and population

**Fig. 1:**
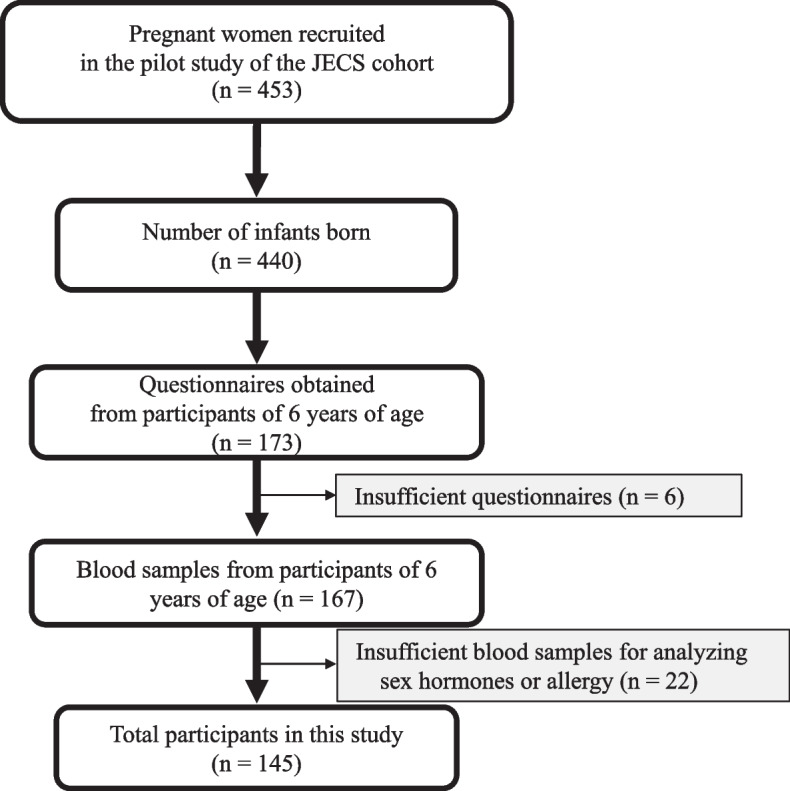
Flowchart of the study population. All participants of the Japan Environment and Children’s Study (JECS) Pilot Cohort are followed until 12 years of age. This study, which was conducted as a part of the JECS Pilot Study, included all 6-year-old children with available samples for the measurement of sex steroid hormone and allergen-specific immunoglobulin levels. Statistical analyses were conducted using previously presented data

The present study included 6-year-old children participating in a pilot birth cohort study in the Japan Environment and Children’s Study (JECS) cohort, which collected data from the questionnaires and blood tests of children at multiple time points during the study [6]. A total of 453 pregnant women from the general population in Japan were recruited for the JECS Pilot Study between February 1, 2009 to March 31, 2010. The JECS Pilot Study included the following four research centers: Jichi Medical University (Tochigi Prefecture), University of Kyusyu (Fukuoka Prefecture), University of Occupational and Environmental Health (Fukuoka Prefecture), and University of Kumamoto (Kumamoto Prefecture). All pregnant women received informed consent, 436 of the 453 pregnant women participated in the JECS Pilot Study, and 440 children were born. As of 2022, the pediatric participants of the ongoing JECS Pilot Study were being continually followed for 12 years. The present study included only the participants who had not received any systemic steroids within 1 month before the blood tests (Fig. 1).

This cross-sectional study evaluated the relationship between sex hormone and allergic diseases at 6 years. The variables used for fitting model were extracted from the questionnaire and medical examination results mentioned above.

### Ethics statement

The JECS Pilot Study was approved by the Institutional Review Board on Epidemiological Studies of the Ministry of the Environment and by the ethics committees of all participating institutions. The JECS Pilot Study was conducted in accordance with the Declaration of Helsinki and other national and international regulations and guidelines. The present study protocol was reviewed and approved by each regional research centers. Informed consent was obtained by all participants’ parents.

### Outcomes variables: allergic disease and allergen-specific IgE titers

#### Allergic disease

Data on allergic diseases (no/yes) were obtained from the questionnaire administered to the caregivers at the time of medical evaluation of children at 6 years of age. Current asthma and Food allergy (FA) were defined as the presence of doctor diagnosed asthma or FA in the past 12 months, and rhinitis was defined as sneezing or runny/congested nose accompanied by itching in the absence of cold or flu in the past 12 months. Atopic dermatitis (AD) was defined based on the U.K. Working Party definition of AD in children, which was diagnosed by a doctor or medical stuff who took a course in a skin observational training session.

The definition of “any allergic disease” was the presence of at least one of the condition, such as current asthma, current rhinitis, AD, or food allergy. For the participants who answered all of the questions about whether they had any of the four allergic disorders, we have also defined the variable “number of allergic diseases” regrouped as 3 levels (0, 1 and ≥ 2).

#### Allergen-specific IgE titers

Allergen-specific IgE titers in serum samples of the participating children at 6 years of age were measured using the densely carboxylated protein microarray (AMERIC, Tokushima, Japan) and were reported as binding unit of IgE (BUe)/mL [7, 8]. Allergen-specific IgE titers were measured for 19 aeroallergen including house dust mite, *Dermatophagoides farinae*, [Der f 1], *Dermatophagoides pteronyssinus*, [Der p 1], dog dander, cat dander, the fungi *Ascomycota*, *Alternaria alternata*: [Alt a 1], *Aspergillus fumigatus*: [Asp f 1], and *Cladosporium herbarum*: [Cla h 8], Japanese cedar, *Cryptomeria japonica*: [Cry j 1], Japanese cypress, birch, alder, timothy grass, sweet vernal grass, orchard grass, and ragweed. The reported limit of aeroallergen-specific IgE was 0.01 BUe/mL for all aeroallergens tested in the current study [7, 9].

### Exposure variables: measurement of sex steroid hormones in serum

In the present study, serum concentrations of total testosterone, estradiol, dehydroepiandrosterone sulfate (DHEA-S), luteinizing hormone (LH), and follicle-stimulating hormone (FSH) were measured in children at 6 years of age. Serum samples were analyzed by an independent clinical contract laboratory.

### Other confounders

The body fat of the participating children at 6 years of age were measured by multi frequency segmental body composition analyzer (MC-780, TANITA, Japan), which calculates muscle mass, body water content, and body fat content at the JECS Pilot Study four research centers at the same day of blood tests. Sex, parental history of allergic disease, and exposure to tobacco smoking at 6 years of age were obtained from questionnaires.

### Statistical analysis

The Mann–Whitney *U* test was used to compare continuous variables, and the χ^2^ test was used to compare categorical variables. Logistic regression analysis was used to evaluate the association of sex steroid hormones with allergic diseases. Covariates adjusted in the models included sex, amount of body fat at 6 years of age, parental history of allergic disease, and exposure to tobacco smoking at 6 years of age. We utilized ordinal logistic regression to build the models for analyzing the relationship between the “number of allergic diseases” and “sex steroid hormones” because the “number of allergic diseases” was an ordinal variable. We also assessed the association of sex steroid hormones with total IgE titers, and some allergens that showed sex differences in distribution. “Total IgE titers” was reclassified as low (< 110 BUe/mL) and high (≥ 110 BUe/mL) levels, and allergen-specific IgE titers were regrouped as low (< 0.01 BUe/mL) and high levels (≥ 0.01 BUe/mL) according to data distribution [10].

As a sensitivity analysis, we also employed the multiple imputations by chained equations (MICE) method to deal with the missing value. The pooled adjusted OR values were estimated using 50 imputed datasets.

The R statistical software version 4.2.3 (Institute for Statistics and Mathematics, Vienna, Austria; www.r-project.org) was used for all statistical analyses, and statistical significance was set at a *P* value of < 0.05.

## Results

### Basic characteristics

The present study included 145 eligible children among a total of 440 children who participated in the JECS Pilot Study (Fig. 1 and Table 1). The study cohort included 74 (51.0%) boys, and the median age was 6.1 (interquartile range [IQR], 6.0–6.1) years. In the entire cohort, 65 (44.8%) had a family history of allergic diseases, 53 (36.6%) children were exposed to secondhand smoke. In the entire cohort, 82 (56.6%) children, including 39 (52.7%) boys and 43 (60.0%) girls, had any allergic diseases (Table E1).

**Table 1:** Features of children in the study

	Number^a^	
Sex, boy, n (%)	145	74 (51.0%)
Age, years, median (first quartile–third quartile)	145	6.1 (6.0–6.1)
Parental allergic history	145	65 (44.8%)
Exposure to tobacco smoking at 6 years of age, n (%)	145	46 (31.7%)
Body fat at 6 years of age, median (kg) (first quartile–third quartile)	139	2.8 (2.0–3.5)
Body fat in boys, median (kg) (first quartile–third quartile)	70	2.7 (2.0–3.6)
Body fat in girls, median (kg) (first quartile–third quartile)	69	2.8 (2.1–3.5)

^a^Number of children without missing values

Sex hormones in serum by gender are presented in Fig. E1. Estradiol and FSH were significantly higher in girls compared to those in boys. The proportion of boy with LH above 0.1 was higher than that in girls. Sex hormones in serum by any allergic diseases are presented in Fig. E2. FSH showed a slight increase in the group of children with allergic diseases.

Figure E3 shows the total and aeroallergen-specific IgE levels. The levels of IgE specific for Dermatophagoides farina, Japanese cedar, Cry j 1, and Japanese cypress were significantly higher in boys than in girls.

### Association between sex hormones in serum and allergic diseases

Table 2 shows the serum sex hormones levels by allergic disease and the ORs from logistic models. After adjusted sex, amount of body fat at 6 years, parental history of allergic disease, and exposure to tobacco smoke, the DHEA-S level was significantly associated with lower odds of any allergic disease (aOR, 0.58; 95%CI, 0.36–0.93; *P* = 0.024) and serum FSH level was significantly associated with higher odds of any allergic disease (aOR 2.04; 95%CI, 1.01–4.11; *P* = 0.046).

**Table 2:** Association between sex hormones in serum and allergic diseases with complete dataset

	Without allergic diseases			With allergic diseases			Logistic regression models^b^			
							aOR	95%CI		*P*
	Median	25^th^	75^th^	Median	25^th^	75^th^		Lower	Upper	
Estradiol, pg/mL^a^	0.22	0.12	1.00	0.28	0.08	1.12	0.67	0.42	1.08	0.10
DHEA-S, pg/mL^a^	75.41	55.45	140.18	73.42	38.54	126.63	0.58	0.36	0.93	0.02
Testosterone, pg/mL^a^	17.90	11.40	24.00	16.20	11.3	24.30	0.81	0.43	1.53	0.52
FSH, mIU/mL^a^	1.30	0.85	1.85	1.60	0.90	2.40	2.04	1.01	4.11	0.05
LH, ≥ 0.1mIU/mL N (%)	10.00 (15.90%)			16.00 (19.80%)			1.94	0.72	5.27	0.19

*Abbreviations*: *aOR* Adjusted odds ratio, *CI* Confidence interval, *DHEA-S* Dehydroepiandrosterone sulphate, *FSH* Follicle-stimulating hormone, *LH* Luteinizing hormone

^a^The exposure variable in the model was log-transformed

^b^All models were adjusted for sex, body fat at age 6, parental history of allergy illness, and smoking exposure

DHEA-S level indicated a significant association with “number of allergic disorders” when the outcome variable was modified to “number of allergic diseases.” The possibility of developing allergy disorders (one or more vs. none) lowers by 44% for every unit increase in log DHEA-S level after adjusting confounders (Table 3).

**Table 3:** Association between sex hormones in serum and number of allergic diseases with the complete dataset

	aOR^a^	95%CI		*P*
		Lower	Upper	
Estradiol, pg/mL^b^	0.77	0.50	1.16	0.21
DHEA-S, pg/mL^b^	0.56	0.37	0.86	0.01
Testosterone, pg/mL^b^	0.70	0.38	1.25	0.23
FSH, mIU/mL^b^	1.65	0.90	3.07	0.11
LH, mIU/mL (≥ 0.1 vs < 0.1)	1.16	0.48	2.81	0.74

*Abbreviations*: *aOR* Adjusted odds ratio, *CI* Confidence interval, *DHEA-S* Dehydroepiandrosterone sulphate, *FSH* Follicle-stimulating hormone, *LH* Luteinizing hormone

^a^The number of allergic diseases was the model’s outcome event and ordinal logistic regression models were constructed. All models were adjusted for sex, body fat at age 6, parental history of allergy illness, and smoking exposure

^b^The exposure variable in the model was log-transformed

### Association between sex hormones in serum and aeroallergen-specific IgE levels

Table 4 represents the relationship between serum sex hormones and IgE levels. Elevated FSH were substantially associated with total IgE titers. High levels of DHEA-S (aOR 0.45, 95%CI: 0.24–0.82) and testosterone (aOR 0.34, 95%CI: 0.15–0.76) were associated with low levels of Japanese cedar IgE.

**Table 4:** Association of sex hormones in serum with total and allergen-specific IgE titers

Allergen-specific IgE titers (BUe/mL)^a^	Sex hormones in serum	aOR^a^	95%CI		*P*
			Lower	Upper	
Total IgE titers (> 110 vs ≤ 110)	Estradiol, pg/mL^b^	1.15	0.71	1.84	0.57
	DHEA-S, pg/mL^b^	0.79	0.50	1.26	0.32
	Testosterone, pg/mL^b^	1.46	0.77	2.79	0.25
	FSH, mIU/mL^b^	2.17	1.04	4.50	0.04
	LH, mIU/mL (≥ 0.1 vs < 0.1)	2.65	0.99	7.07	0.05
Dermatophagoides farinae (> 0.01 vs ≤ 0.01)	Estradiol, pg/mL^b^	0.74	0.45	1.20	0.22
	DHEA-S, pg/mL^b^	0.70	0.43	1.16	0.17
	Testosterone, pg/mL^b^	0.57	0.28	1.14	0.11
	FSH, mIU/mL^b^	1.38	0.67	2.85	0.38
	LH, mIU/mL (≥ 0.1 vs < 0.1)	0.38	0.13	1.10	0.08
Japanese cedar (> 0.01 vs ≤ 0.01)	Estradiol, pg/mL^b^	0.87	0.51	1.50	0.62
	DHEA-S, pg/mL^b^	0.45	0.24	0.82	0.01
	Testosterone, pg/mL^b^	0.34	0.15	0.76	0.01
	FSH, mIU/mL^b^	0.91	0.42	1.99	0.82
	LH, mIU/mL (≥ 0.1 vs < 0.1)	1.56	0.55	4.44	0.41
Cry j 1 (> 0.01 vs ≤ 0.01)	Estradiol, pg/mL^b^	1.12	0.69	1.81	0.66
	DHEA-S, pg/mL^b^	0.90	0.56	1.47	0.68
	Testosterone, pg/mL^b^	0.76	0.38	1.49	0.42
	FSH, mIU/mL^b^	0.79	0.38	1.63	0.52
	LH, mIU/mL (≥ 0.1 vs < 0.1)	0.83	0.30	2.34	0.73
Japanese cypress (> 0.01 vs ≤ 0.01)	Estradiol, pg/mL^b^	1.04	0.63	1.70	0.89
	DHEA-S, pg/mL^b^	0.66	0.40	1.11	0.12
	Testosterone, pg/mL^b^	0.55	0.27	1.12	0.10
	FSH, mIU/mL^b^	0.60	0.29	1.25	0.17
	LH, mIU/mL (≥ 0.1 vs < 0.1)	0.99	0.36	2.71	0.99

*Abbreviations*: *aOR* Adjusted odds ratio, *CI* Confidence interval, *DHEA-S* Dehydroepiandrosterone sulphate, *FSH* Follicle-stimulating hormone, *LH* Luteinizing hormone

^a^Outcome variable in the models was reclassified as a dichotomous variable (high level was outcome event), and logistic regression models were fitted. All models were adjusted for sex, body fat at age 6, parental history of allergy illness, and smoking exposure

^b^The exposure variable in the model was log-transformed

### Sensitive analysis

The pooled aORs from the multiple imputation approach (Table E2) matched those from the full dataset (Tables 2 and 3). High DHEA-S levels were associated with a decreased risk of allergy disorders after controlling for covariates (aOR: 0.62; 95%CI: 0.39–0.98 in the logistic regression model and 0.61 (0.40–0.92) in the ordinal logistic regression models).

## Discussion

In the present study, which included 145 six-year-old Japanese children participating in the JECS Pilot Study, we found that higher serum DHEA-S level was significantly associated with decreased odds of any allergic disease Furthermore, DHEA-S level showed a significant association with number of allergic diseases. We discovered an association between high DHEA-S and testosterone levels and reduced IgE levels in Japanese cedar. To our knowledge, this is the first study to examine the relationship between Japanese children’s levels of sex-steroid hormones and allergic diseases.

In DHEA-S, there are several reports suggesting association with prevalence of the allergic disease. A cross-sectional study of children examining the association of sex steroid hormones with asthma, including 45 boys and 23 girls between the ages of 6 and 18 years reported that serum DHEA-S levels were negatively associated with asthma symptoms and were positively associated with forced expiratory volume in one second and forced vital capacity in boys. Conversely, serum estradiol levels were negatively associated with forced expiratory volume in one second and forced vital capacity in girls [11]. Another study found that serum levels of DHEA and testosterone were lower in male patients with AD compared to the reference group of males [12]. Lower serum levels of DHEA and DHEA-S irrespective of sex were also reported in patients with asthma or chronic spontaneous urticaria compared to the reference group [13]. The results of this study on serum DHEA-S levels agree with previous studies examining the association of sex steroid hormones with allergic diseases.

Sex hormone association with immune responses, such as IgE production and related allergic reactions, were reported in animal models. In mice, the levels of immunoglobulins, including IgM, IgG1, IgG2, and IgE, were higher in female than male. Moreover, estrogen inhibitor injection to the female mice have reduced the levels of allergen-specific IgG1, IgG2a, and IgE levels and attenuate clinical allergic disease symptoms [14]. Another report revealed that estrogen treatment in male mice has been shown to upregulate immunoglobulin levels and to induce antigen-specific antibody-producing cells [15, 16]. DHEA and testosterone are also known to have a significant relationship with allergic inflammation. In comparison to control male mice, eosinophil and lymphocyte infiltration caused by OVA, as well as the production of the IL-13 protein increased significantly in castrated male mice due to the reduction of testosterone. Additionally, when DHEA was injected into castrated male mice, the allergic airway inflammation of the mice was reduced in comparison to the control mice [17]. Furthermore, mice sensitized on house dust mite treated with DHEA had lower serum levels of eosinophils, IL-5, IL-4, and IFN-g but no change in serum IgE concentrations, compared to mice sensitized on house dust mite without DHEA treatment [17]. These reports indicate that estrogen may increase type 2 T helper cell (Th2)-mediated allergic inflammation and DHEA and testosterone may decrease the Th2-mediated allergic inflammation. Therefore, we think sex hormones, such as estrogen, testosterone, and DHEA-S, were important in regulating type 2 inflammation and many pathways are affected by ovarian hormone and/or testosterone signaling. Several studies in animal models have reported that allergen-induced, Th2-mediated allergic inflammation is promoted by estrogen signaling and attenuated by androgen signaling [18–20]. Sex hormones could be involved in the regulation of the immune response, and this study adds evidence to this speculation. However, further confirmation is required by carrying out studies with a large sample size.

We also found that FSH was associated with higher odds of any allergic disease. Intriguingly, a study reported that the baseline FSH levels were higher in patients with premenstrual exacerbation of asthma than patients without exacerbation [21]. Although studies examining the association of FSH with allergic diseases are limited, it is possible that FSH might indirectly contribute to the increased prevalence of allergic diseases by upregulating the levels of hormones such as estrogen and progesterone, thereby enhancing Th2-mediated allergic inflammation.

Our study has several limitations. First, due to a relatively long time since the allergic sickness, parents may purposefully or inadvertently underreport allergic diseases in their children on the questionnaire. Second, the JECS cohort study measures sex hormone levels in participants aged 6 years or older, and the temporal relationships were not evaluated as data on older children were not yet available in the ongoing study. Third, the sample size might be small to evaluate the significant association between allergic diseases and sex. The ongoing JECS cohort will continue following the children included in the current study to examine the association between sex steroid hormones and allergic diseases.

## Conclusions

Our findings suggest that sex hormones might play an important role in development of allergic diseases observed before puberty. Further studies are needed to elucidate the regulatory roles of sex hormones in allergy development, the mechanisms underlying inflammatory responses in allergic diseases, and the regulation of systematic inflammation for the development of hormonal therapeutic approaches in allergic diseases. The ongoing JECS cohort will continue following these children to examine changes in the association between sex steroid hormones and allergic diseases.

### Supplementary Information

**Additional file 1: Table E1.** Rates of specific allergic diseases. **Table E2.** Association between sex hormones in serum and any allergic diseases/number of allergic diseases with multiple imputation. **Figure E1.** Sex hormones in serum by sex. A: Testosterone; B: Estradiol; C: DHEA-S; D: FSH; E: LH; (****: *p* < 0.0001, ns: *p* > 0.05). DHEA-S, dehydroepiandrosterone sulphate; FSH, follicle-stimulating hormone; LH, luteinizing hormone. **Figure E2.** Sex hormones in serum by any allergic disease. A: Testosterone; B: Estradiol; C: DHEA-S; D: FSH; E: LH; (*: *p* < 0.05, ns: *p* > 0.05). DHEA-S, dehydroepiandrosterone sulphate; FSH, follicle-stimulating hormone; LH, luteinizing hormone. **Figure E3.** Levels of allergen-specific IgE titers in boys and girls.

## Data Availability

Data are unsuitable for public deposition due to ethical restrictions and legal framework of Japan. It is prohibited by the Act on the Protection of Personal Information (Act No. 57 of 30 May 2003, amendment on 9 September 2015) to publicly deposit the data containing personal information. Ethical Guidelines for Medical and Health Research Involving Human Subjects enforced by the Japan Ministry of Education, Culture, Sports, Science and Technology and the Ministry of Health, Labour and Welfare also restricts the open sharing of the epidemiologic data. All inquiries about access to data should be sent to: jecs-en@nies.go.jp. The person responsible for handling enquiries sent to this e-mail address is Dr. Shoji F. Nakayama, JECS Programme Office, National Institute for Environmental Studies.

## Abbreviations

DHEA-S: Dehydroepiandrosterone sulfate
FSH: Follicle-stimulating hormone
JECS: Japan Environment and Children’s Study
