# Supplementary material for: Sex steroid hormones and allergic diseases in children: a pilot birth cohort study in the Japan Environment and Children’s Study cohort
Source: BMC Pediatr. 2023 Sep 21;23:479. doi: 10.1186/s12887-023-04302-9 (PMC10512488; doi:10.1186/s12887-023-04302-9)
Supplement: Supplementary file 1 — Additional file 1: Table E1. Rates of specific allergic diseases. Table E2. Association between sex hormones in serum and any allergic diseases/number of allergic diseases with multiple imputation. Figure E1. Sex hormones in serum by sex. A: Testosterone; B: Estradiol; C: DHEA-S; D: FSH; E: LH; (****: p < 0.0001, ns: p > 0.05). DHEA-S, dehydroepiandrosterone sulphate; FSH, follicle-stimulating hormone; LH, luteinizing hormone. Figure E2. Sex hormones in serum by any allergic disease. A: Testosterone; B: Estradiol; C: DHEA-S; D: FSH; E: LH; (*: p < 0.05, ns: p > 0.05). DHEA-S, dehydroepiandrosterone sulphate; FSH, follicle-stimulating hormone; LH, luteinizing hormone. Figure E3. Levels of allergen-specific IgE titers in boys and girls. [file 12887_2023_4302_MOESM1_ESM.docx]

**Supplement files**

**Table E1** Rates of specific allergic diseases.

**Table E2** Association between sex hormones in serum and any allergic diseases/number of allergic diseases with multiple imputation

**Figure E1** Sex hormones in serum by sex. A: Testosterone; B: Estradiol; C: DHEA-S; D: FSH; E: LH; (****: p<0.0001, ns: p>0.05). DHEA-S, dehydroepiandrosterone sulphate; FSH, follicle-stimulating hormone; LH, luteinizing hormone.

**Figure E2** Sex hormones in serum by any allergic disease. A: Testosterone; B: Estradiol; C: DHEA-S; D: FSH; E: LH; (*: p<0.05, ns: p>0.05). DHEA-S, dehydroepiandrosterone sulphate; FSH, follicle-stimulating hormone; LH, luteinizing hormone.

**Figure E3** Levels of allergen-specific IgE titers in boys and girls

**Table E1** Rates of specific allergic diseases in the study

|  |  | ALL |  |  |  | Boys |  |  |  | Girls |  |  |
| --- | --- | --- | --- | --- | --- | --- | --- | --- | --- | --- | --- | --- |
|  | n | N | % |  | n | N | % |  | n | N | % | p |
| Any allergic disease | 82 | 145 | 56.6 |  | 39 | 74 | 52.7 |  | 43 | 71 | 60.6 | 0.403 |
| Current asthma | 16 | 145 | 11.0 |  | 7 | 74 | 9.5 |  | 9 | 71 | 12.7 | 0.603 |
| Atopic dermatitis | 47 | 145 | 32.4 |  | 22 | 74 | 29.7 |  | 25 | 71 | 35.2 | 0.595 |
| Current rhinitis | 47 | 142 | 33.1 |  | 21 | 72 | 29.2 |  | 26 | 70 | 37.1 | 0.373 |
| Food allergy | 10 | 145 | 6.9 |  | 6 | 74 | 8.1 |  | 4 | 71 | 5.6 | 0.746 |
| Number of allergic diseases |  |  |  |  |  |  |  |  |  |  |  |  |
| 0 | 62 | 142 | 43.7 |  | 34 | 72 | 47.2 |  | 28 | 68 | 41.2 |  |
| 1 | 52 | 142 | 36.6 |  | 24 | 72 | 33.3 |  | 28 | 68 | 41.2 |  |
| ≥2 | 28 | 142 | 19.7 |  | 14 | 72 | 19.4 |  | 12 | 68 | 17.6 |  |

**Table E2** Association between sex hormones in serum and any allergic diseases/number of allergic diseases with multiple imputation

|  | Logistic regression models^&^ | | | |  | Ordinal Logistic regression models^$^ | | | |
| --- | --- | --- | --- | --- | --- | --- | --- | --- | --- |
|  |  | 95%CI | |  |  |  | 95%CI I | |  |
|  | aOR | Lower | Upper | P |  | aOR | Lower | Upper | P |
| Estradiol, pg/mL^#^ | 0.68 | 0.42 | 1.08 | 0.105 |  | 0.78 | 0.51 | 1.18 | 0.239 |
| DHEA-S, pg/mL^#^ | 0.62 | 0.39 | 0.98 | 0.043 |  | 0.61 | 0.40 | 0.92 | 0.021 |
| Testosterone, pg/mL^#^ | 0.89 | 0.48 | 1.66 | 0.714 |  | 0.79 | 0.44 | 1.39 | 0.408 |
| FSH, mIU/mL^#^ | 2.05 | 1.03 | 4.08 | 0.043 |  | 1.71 | 0.94 | 3.11 | 0.082 |
| LH, mIU/mL (≥0.1 vs <0.1) | 1.79 | 0.68 | 4.67 | 0.238 |  | 1.25 | 0.53 | 2.94 | 0.617 |

Abbreviations: aOR, adjusted odds ratio; CI, confidence interval; DHEA-S, dehydroepiandrosterone sulphate; FSH, follicle-stimulating hormone; LH, luteinizing hormone.

^#^ The exposure variable in the model was log-transformed.

^&^ Outcome event in the models was allergic diseases.

^$^ Outcome event in the models was numbers of allergic diseases.

All models were adjusted by sex, amount of body fat at 6 years, parental history of allergic disease, and exposure to tobacco smoke.


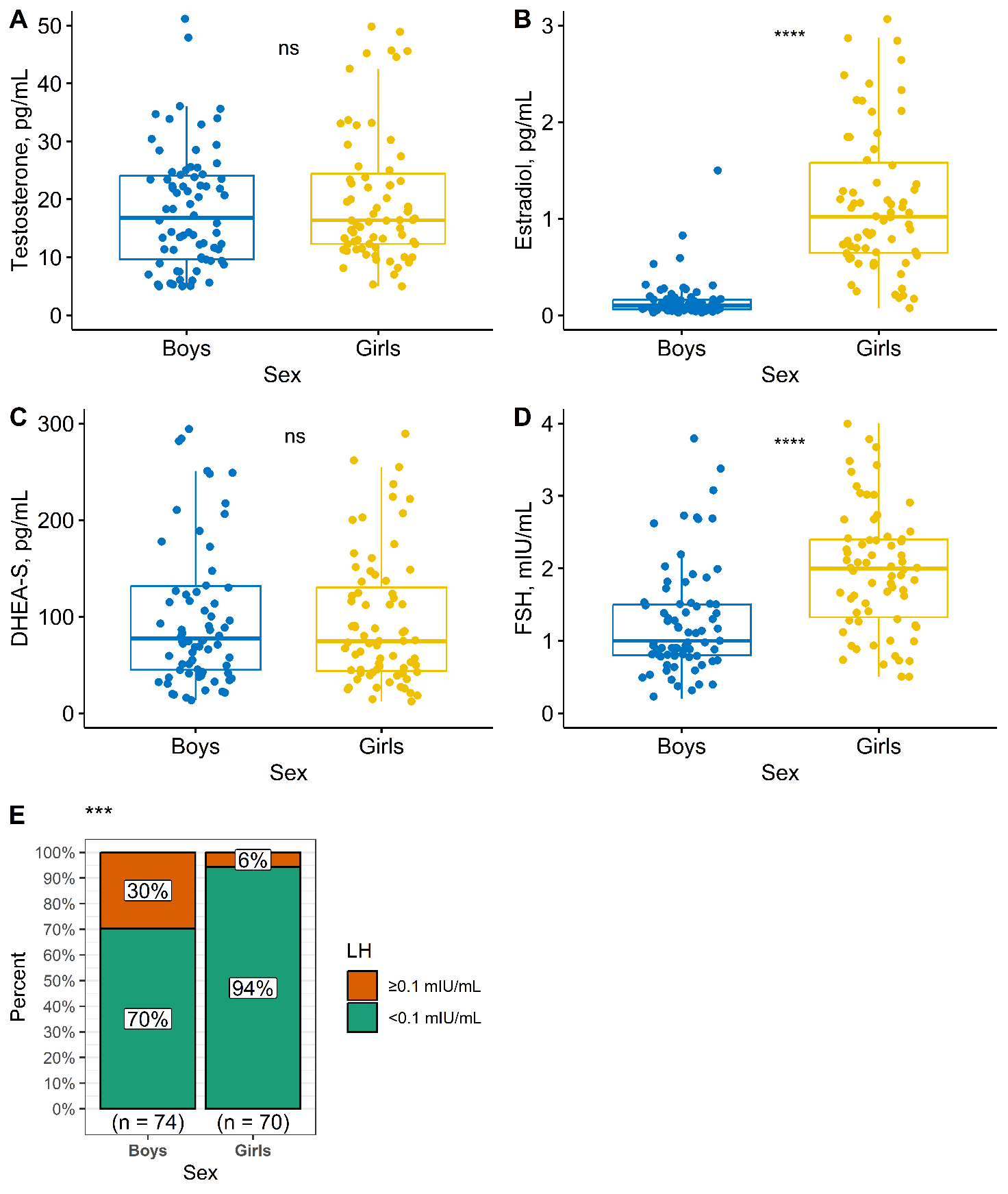


Figure E1


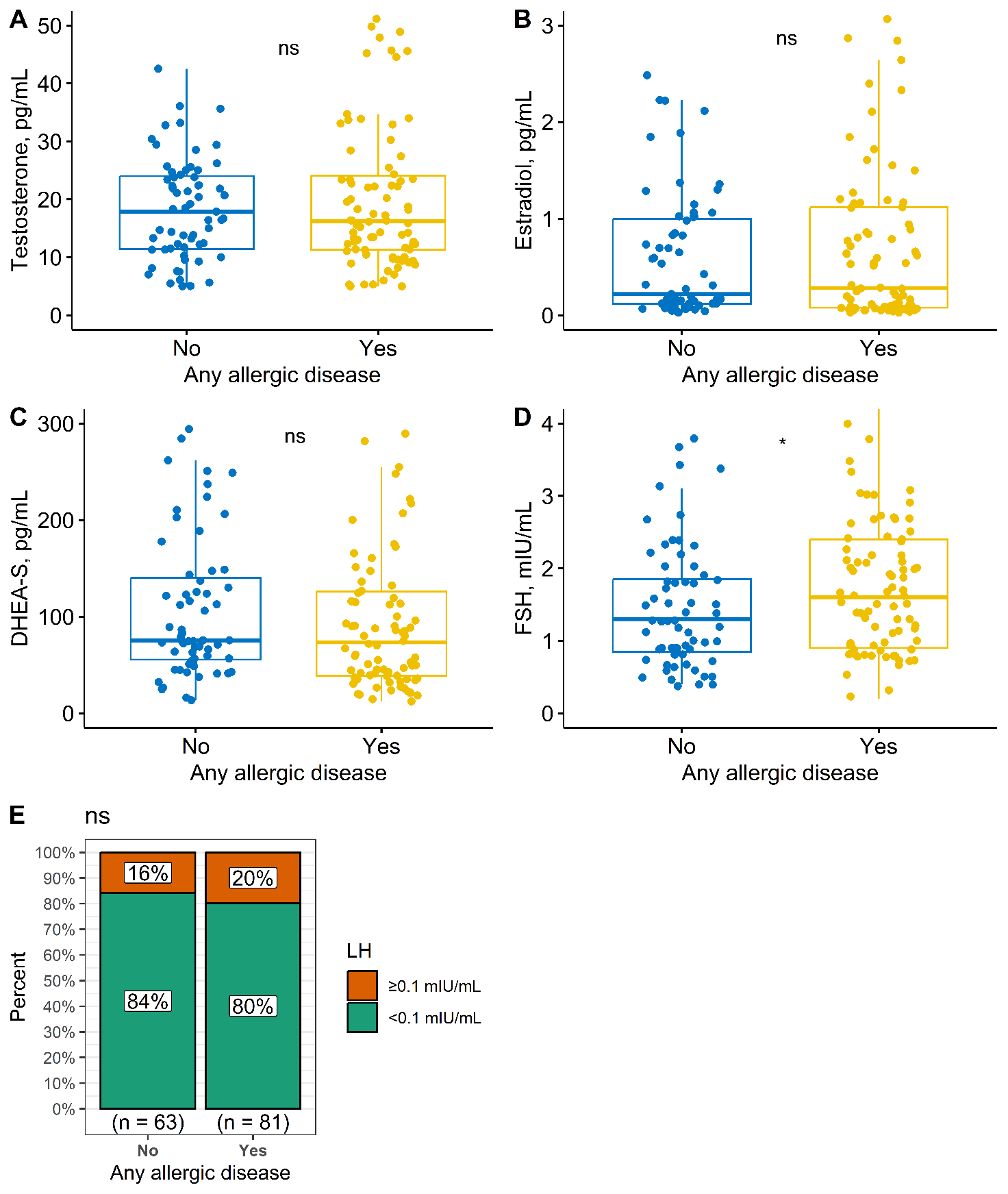


Figure E2


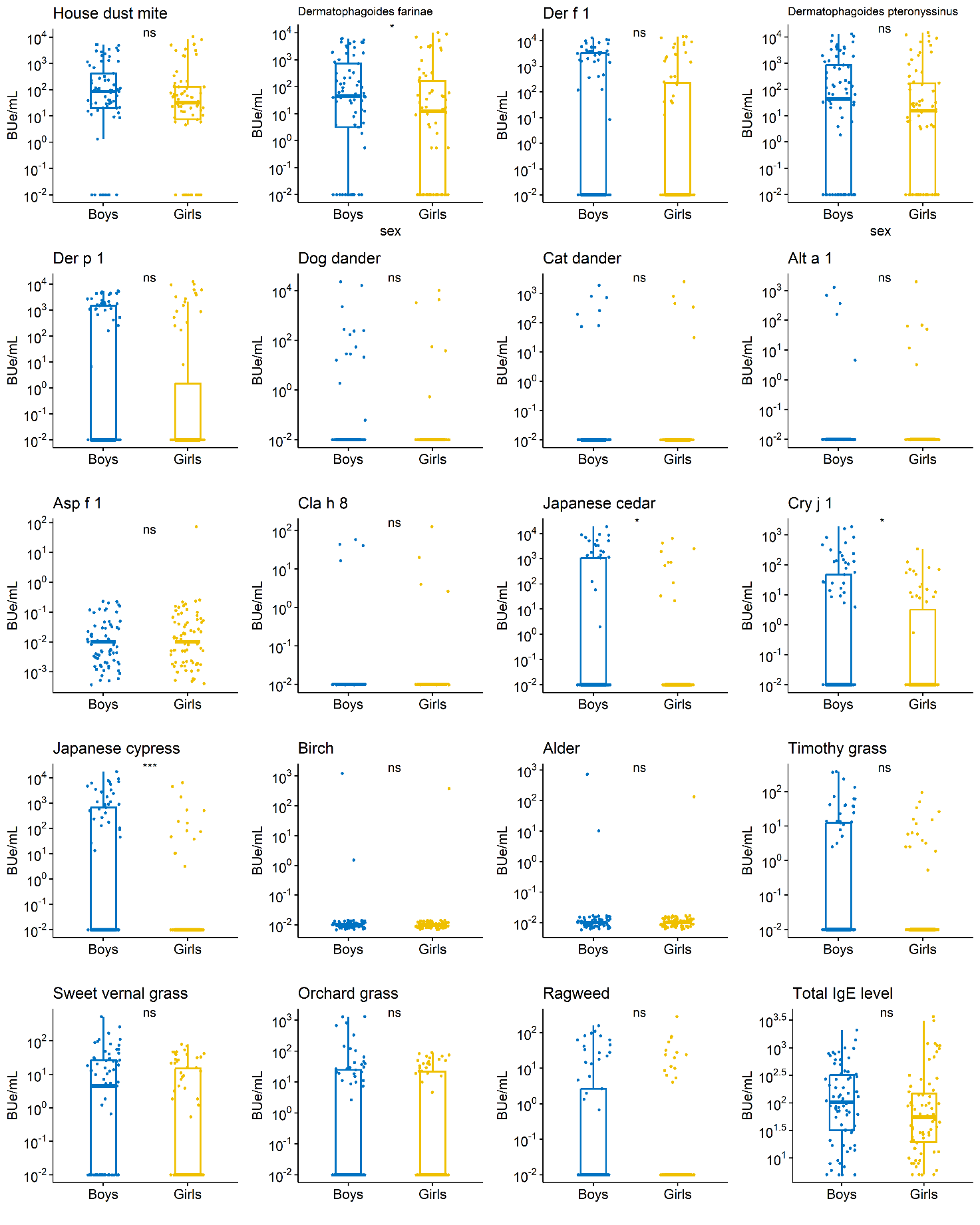


Figure E3
